# Driving Waveform Design of Electrophoretic Display Based on Optimized Particle Activation for a Rapid Response Speed

**DOI:** 10.3390/mi11050498

**Published:** 2020-05-14

**Authors:** Wenyao He, Zichuan Yi, Shitao Shen, Zhenyu Huang, Linwei Liu, Taiyuan Zhang, Wei Li, Li Wang, Lingling Shui, Chongfu Zhang, Guofu Zhou

**Affiliations:** 1 College of Electron and Information, University of Electronic Science and Technology of China, Zhongshan Institute, Zhongshan 528402, China; wenyao.he@guohua-net.com (W.H.); Shuill@m.scnu.edu.cn (L.S.); cfzhang@uestc.edu.cn (C.Z.); 2 Institute of Electronic Paper Displays, South China Academy of Advanced Optoelectronics, South China Normal University, Guangzhou 510006, China; shenshitao@m.scnu.edu.cn (S.S.); 2018023170@m.scnu.edu.cn (Z.H.); yoeksome@sina.com (L.L.); taiyuan.zhang@guohua-oet.com (T.Z.); Wei.li@guohua-oet.com (W.L.); creekxi@163.com (L.W.); guofu.zhou@m.scnu.edu.cn (G.Z.); 3 Shenzhen Guohua Optoelectronics Tech. Co., Ltd., Shenzhen 518110, China

**Keywords:** electrophoretic display, driving waveform, particle activation, response speed, reference grayscale

## Abstract

Electrophoretic displays (EPDs) have excellent paper-like display features, but their response speed is as long as hundreds of milliseconds. This is particularly important when optimizing the driving waveform for improving the response speed. Hence, a driving waveform design based on the optimization of particle activation was proposed by analyzing the electrophoresis performance of particles in EPD pixels. The particle activation in the driving waveform was divided into two phases: the improving particle activity phase and the uniform reference grayscale phase. First, according to the motion characteristics of particles in improving the particle activity phase, the real-time EPD brightness value can be obtained by an optical testing device. Secondly, the derivative of the EPD brightness curve was used to obtain the inflection point, and the inflection point was used as the duration of improving particle activity phase. Thirdly, the brightness curve of the uniform reference grayscale phase was studied to set the driving duration for obtaining a white reference grayscale. Finally, a set of four-level grayscale driving waveform was designed and validated in a commercial E-ink EPD. The experimental results showed that the proposed driving waveform can cause a reduction by 180 ms in improving particle activity phase and 120 ms in uniform reference grayscale phase effectively, and a unified reference grayscale can be achieved in uniform reference grayscale phase at the same time.

## 1. Introduction

Electrophoretic display (EPD) technology has been a research topic of interest for many years due to its wide market potential [1]. As a paper-like display technology, EPDs have the advantages of paper-like display effect, ultra-low power consumption, and being readable under bright light [2,3]. In recent years, EPDs have been widely used in e-books, electronic labels, and smart watches, amongst others [4,5]. The grayscale display of an EPD depends on the distribution of black and white particles in a microcapsule, which can be driven by voltage timing applied on the pixel, and the voltage timing is called the driving waveform. As a core part of EPDs, the length of the driving waveform can affect the response speed of EPDs directly. Therefore, it is of great significance to improve the response speed and reduce visibility of the ghost image of EPDs by optimizing the driving waveform design [6]. 

Currently, the driving waveform is divided into three phases: erase original image phase, particle activation phase, and new image phase [7]. In driving processes, the photoelectric properties of the microcapsule system are nonlinear, and it is difficult to form a grayscale display accurately [8]. A lot of time is required to unify the spatial position of particles for driving to a target grayscale, and this process takes hundreds of milliseconds, or even one second [9]. The erase original image phase can obtain a stable state which is in a white or black state. The new image phase is used for driving the EPD to a new grayscale [10]. When a particle has been kept at the same state for a long period, the mobility of charged particles becomes much lower than others. The particle activity can be improved by driving the panel several times between two optical extremes (black/white extremes states) in the particle activation phase [11,12]. In EPDs, the distribution of individual black or white particles is random, so it is necessary to find a relatively stable grayscale as a reference grayscale. The white grayscale is usually used as the reference grayscale, and the display of other grayscale are obtained by using the white grayscale as the driving start point. This driving mode has been used up to now, and plays an important role in EPD products [13]. However, the particle activation phase is longer than the other two phases and has a significant impact on the response speed of EPDs. In order to optimize the activation phase, the particle activation can be optimized by a high-frequency voltage mode, but it is difficult to obtain a stable reference grayscale and the driving duration can reach 960 ms [14]. Some scholars have removed the particle activation phase and added a response latency to eliminate the original image, but the reference grayscale uniformity cannot be obtained, resulting in ghosting after writing new images many times [15]. At the same time, some short waveforms have been proposed, which were divided into three phases: zero voltage bias phase, high-frequency activation phase, and driving phase [16,17], but with reduced display quality of EPDs [18,19]. 

By analyzing the electrophoresis performance of particles, a driving waveform design of EPDs based on optimized particle activation phase for a rapid response speed was proposed in this study. The activation phase of the driving waveform was divided into the improving particle activity phase and the uniform reference grayscale phase. In one phase, the derivative of the EPD brightness curve was used to obtain the inflection point, which was used as the duration of improving particle activity phase. In the other phase, the brightness curve of the uniform reference grayscale phase was studied to set the driving duration for obtaining a white reference grayscale. Currently, the designed waveforms have been validated in commercial E-ink EPDs. This can shorten the driving duration, improve the response speed, and reduce visibility of the ghost image effectively. 

## 2. System Design Principle

### 2.1. Display Principle

**Figure 1:**
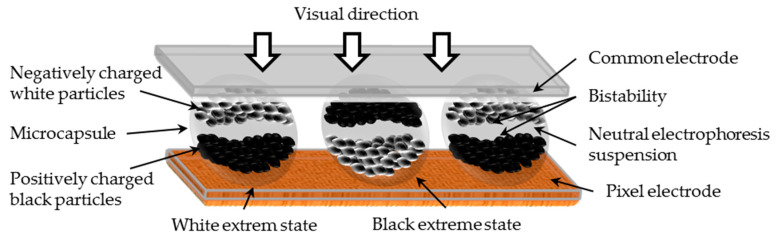
Schematic of an electrophoretic display (EPD) structure. The bottom is the pixel electrode, the middle is the microcapsule which includes a neutral electrophoresis suspension and white and black electrophoretic particles, while the top is the common electrode. The spatial position of particles cannot be changed after the removal of the pixel electrode voltage, which is referred to as bistability.

The motion and distribution of electrophoretic particles in microcapsules is very complex [20]. The trajectories of charged particles interfere with each other, and the attraction and repulsion among particles exist at the same time [21]. During a grayscale display process in EPDs, the spatial position of the particles contained in the microcapsule determines the grayscale value in a pixel. The positively charged black particles can move toward the common electrode by the force of the electric field when a positive voltage is applied to the pixel electrode, and the negatively charged white particles can move toward the pixel electrode such that the display observed by the human eye is black, as shown in Figure 1 [22]. When all of the black particles are driven to the common electrode, the state is referred to as the black extreme state. The white particles move to the common electrode when a negative voltage is applied to the pixel electrode, and the display observed by the human eye is white [23]. When all of white particles have been driven to the common electrode, the state is referred to as the white extreme state. The voltage sequence applied on pixels is called the driving waveform, and its quality directly determines the display effect of EPDs. The internal electrophoretic particles cannot be displaced when the electric field is not applied to the pixel, which is referred to as bistability EPDs [24]. 

With the driving of the electric field, the charged particles are subjected to electric field forces as Equation (1):

(1) $$F_{c}=q\times E$$

where $F_{c}$ is an electric field force, q is the charge of a particle, and E is the electric field intensity. The particle motion is hampered by Stokes forces when particles are driven in the liquid, and the expression is shown in Equation (2):

(2) Fd=6πμvr

where Fd is Stokes force, μ is liquid viscosity coefficient, v is the motion relative rate between particles and fluids, and r is sphere particle diameter. In addition, the number of charges which are carried by a particle is 50–100 when the particle radius is within 1 μm. The materials in microcapsules include insulating oil, electrophoretic particles, and density balancing agent [25]. The parameters related to electrophoretic phenomena are particles mass and zeta potential, dielectric properties, viscosity, and electric field strength of the insulating oil. The particles can be driven and are in an accelerated motion state when the electric field force is greater than the Stokes force, and the combined force is shown in Equation (3):

(3) $$F=Fc-Fd=m\frac{dv}{dt}$$

where *m* is the mass of a particle and $\frac{dv}{dt}$ is the acceleration. The differential equation of Equation (3) is shown as Equation (4) [26]:

(4) $$vi=\frac{qE}{6\pi \mu V}[1\pm \exp(-\frac{6\pi \mu V}{m}t)]$$

where vi is the velocity of a particle, which is proportional to the electric field strength. The particle’s motion distance can be obtained by Equation (5):

(5) s=vi×t

where t is the particle’s driving time and *s* is the particle’s motion distance, which can be reflected by the change in EPD reflectivity. According to the CIELab (International Committee on Illuminate Lab color space) standard, the relationship between brightness and reflectivity can be calculated as Equation (6):

(6) $$L^{*}=116\times (\sqrt[{3}]{\frac{R}{R0}})-16$$

where R is the reflectivity of an EPD, R0 is the reference standard of 100% reflectivity, and $L^{*}$ is the brightness of the EPD. The brightness value error of EPDs are mainly caused by the ghost image [27], as shown in Equation (7) [28]:

(7) $$\Delta L^{*}{}_{GS(n)}=L^{*}{}_{GS(n),Max}-L^{*}{}_{GS(n),Min}$$

where $\Delta L^{*}{}_{GS(n)}$ is the ghost value, $L^{*}{}_{GS(n),Max}$ is the maximum brightness value of EPDs, $L^{*}{}_{GS(n),Min}$ is the minimum brightness value of EPDs, and *n* is the grayscale level. The uniformity of the grayscale needs to be detected when the image is refreshed. By using the gray differential statistics, a grayscale image is expressed as a function H(x,y), and (x,y) is a point in the image. (x+Δx,y+Δy) is the adjacent point of (*x*,*y*), and the gray difference between the two points is shown as Equation (8):

(8) $$H_{\Delta }(x,y)=H(x,y)-H(x+\Delta x,y+\Delta y)$$

where $H_{\Delta }$ is the grayscale difference. The probability of getting each grayscale differential is p(i). Equation (9) is used to calculate the texture feature value entropy *e* in a grayscale image, and texture is a phenomenon of nonuniform grayscale display in an EPD. The nonuniformity of the texture in the grayscale image is reflected, and the smaller the value, the more even the texture in the grayscale image, where the expression is shown in Equation (9):

(9) $$e=-\sum_{i=0}^{n}p(i)\log_{2}[p(i)]$$

### 2.2. Design Principles for Driving Waveforms

**Figure 2:**
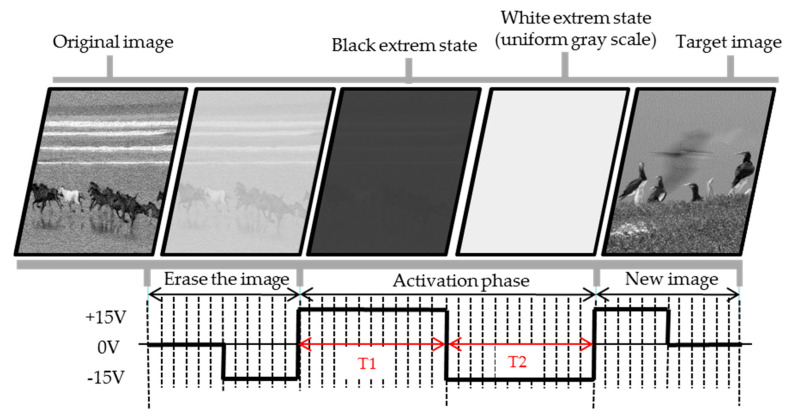
A typical EPD conventional driving waveform contains three phases for image updating. The T1 phase is used as improving particle activity phase and the T2 phase is used as uniform reference grayscale phase in the activation phase. The image in T1 is erased in T2.

The characteristics of conventional driving waveform are shown in Figure 2. It is usually divided into three phases: erase the image, activation phase, and new image phase. The driving durations of three phases are 240, 480, and 240 ms, respectively. 

In the original image, the particle spatial position in each pixel is different. Hence, there are different grayscale values in each pixel when the driving voltage is the same as each other. It’s easy to form a ghost image, and the EPD can be driven to a uniform optical extremes states (black or white extremes states) for eliminating ghost images, which is called as the erase the image phase. 

In addition, the longer a pixel remains in the same state, the lower the mobility of charged particles, which can hinder the driving of a new grayscale in EPDs. By driving the display several times between two optical extremes, it can improve the activity of particles and further eliminate ghosts, and this phase is called as particle activation phase. In this study, the particle activation phase was divided into two phases: improving particle activity phase and uniform reference grayscale phase (white extreme state). The main function of the improving particle activity phase is to increase particle activity, which can improve the particle driving speed. Then, the main function of the uniform reference grayscale phase is to unify a reference grayscale, so as to ensure that particles can drive to a same spatial position form different initial grayscale before new image phase. 

In the new image phase, the target grayscale is produced in the end of pulse sequence. Different driving voltages with different driving durations are applied to different pixels for driving different grayscale. These three phases are combined into a complete driving waveform, and the n-level grayscale requires n^2^ driving waveforms, then multilevel grayscale driving waveforms are combined into a lookup table [29]. In the display process, the driving waveform can be called by the driver system according to different initial grayscale and target grayscale. 

## 3. Design of the Activation Phase in Driving Waveforms

The particle motion characteristics in the improving particle activity phase were studied by us according to the change of display brightness value. The inflection point of particles brightness was obtained by solving the first derivative of the brightness curve, which was the duration of the improving particle activity phase. The driving duration for the uniform reference grayscale phase was then studied to shorten the driving waveform. 

### 3.1. Response Time Characteristics of the Improving Particle Activity Phase

**Figure 3:**
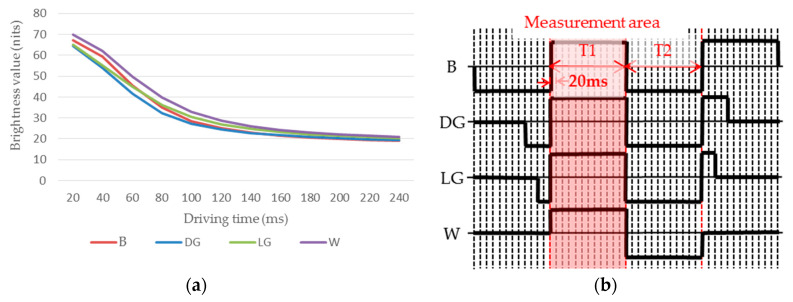
Brightness change curve of the improving particle activity phase at different initial grayscale. (**a**) The brightness curve of the T1. (**b**) The driving waveform used in measurements.

The brightness changes in improving particle activity phase of driving waveforms for a four-level grayscale were measured, and black (B), dark gray (DG), light gray (LG), and white (W) were set as the initial grayscale, as shown in Figure 3. 

**Figure 4:**
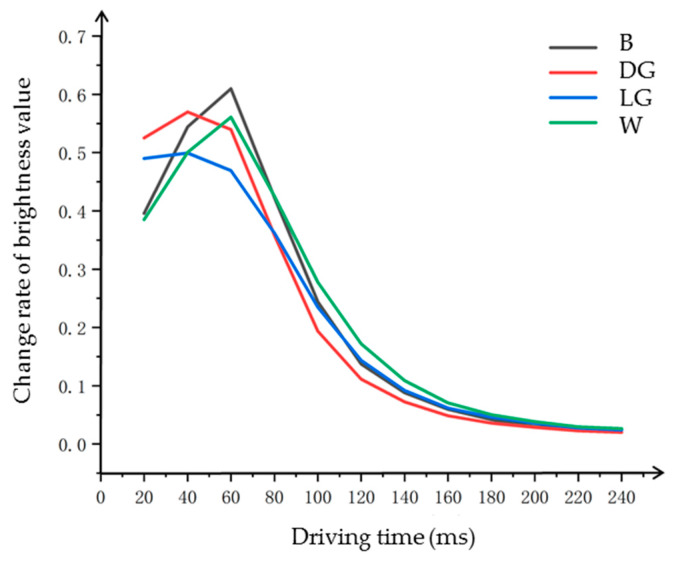
First order derivative curve of brightness value in improving particle activity phase. “B”, “DG”, “LG”, and ”W” indicate different initial grayscale in the driving waveform.

The initial brightness values of the four different initial grayscale are not the same, but the brightness values can be closed together after the image erasure phase in the driving waveform (the phase before T1). Hence, when the measuring area is T1 phase, the brightness value is consistent. The motion speed of particles is directly proportional to the change speed of the display brightness value. The motion of particles is shown as the brightness change of EPDs. In Figure 3a, it can be seen that the brightness value decreases gradually, and the speed of the decrease rate increased and then decreased, so the motion speed of particles was increased and then decreased. In order to calculate the change of particles motion speed, the first order derivative was carried out for each curve, as shown in Figure 4. 

As can be seen, the change rate of the EPD brightness was the highest when the time was 40–60 ms, when the particle motion had a highest speed. Thus, the particle activity was highest when the driving duration of T1 was 40–60 ms, which was conducive for driving effect in the next phase.

### 3.2. Response Time Characteristics of the Uniform Reference Grayscale Phase

**Figure 5:**
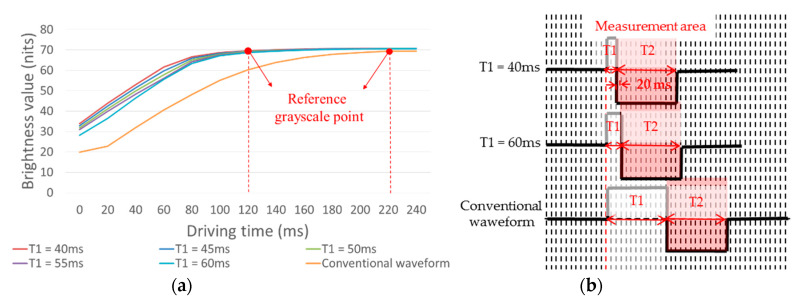
The influence of T1 timing on T2. (**a**) The brightness curve of T2. (**b**) The driving waveform used in the measurement.

The brightness change value of T2 was measured when the white grayscale was set as the initial grayscale. As shown in Figure 5, the driving duration of T1 was 40, 45, 50, 55, and 60 ms, respectively. 

As shown in Figure 5a, the time required for reaching the reference grayscale is 120 ms when T1 is 40, 45, 50, 55, and 60 ms. In the conventional driving waveform, the time required for reaching the reference grayscale is 220 ms. The change in brightness value of T2 is shown in Table 1. 

**Figure 6:**
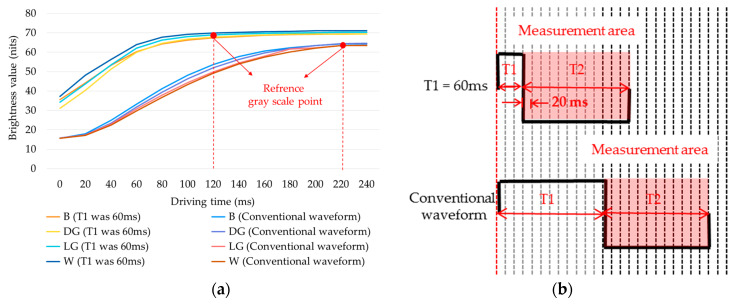
Brightness curves at different initial grayscale. (**a**) The brightness curve of T2. (**b**) The driving waveform used in this measurement.

The initial brightness of the EPD was more than 30 nits, when the T1 was 40, 45, 50, and 55 ms respectively, which showed that the EPD cannot reach optical extremes (black or white states) when the driving duration of 40, 45, 50, and 55 ms was applied continuously in T1, and it was not conducive to the particle driving in the next phase [30]. Therefore, 40, 45, 50, and 55 ms were not suitable for setting as the duration of T1. The driving duration to stable reference grayscale was 120 ms when T1 was 60 ms, and the driving duration to a stable reference grayscale was 220 ms when T1 was 240 ms in the conventional driving waveform, which showed that it was easier to obtain a stable reference grayscale with the uniform reference grayscale phase than the conventional driving waveform. The curve of brightness when T1 was 60 ms and the conventional driving waveform are shown in Figure 6. 

### 3.3. Four-Level Grayscale Driving Waveform Design

**Figure 7:**
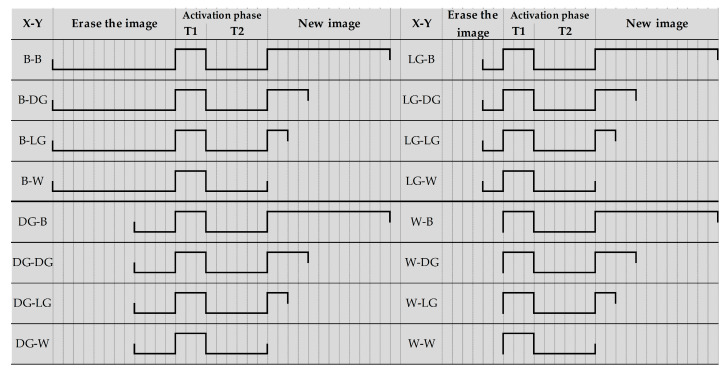
A four-level grayscale driving waveform set. X is displayed as an original grayscale, and Y can be displayed when the EPD has been driven by the driving waveform.

In this paper, a four-level grayscale driving waveform was taken as an example to test the performance of the driving waveform. The proposed driving waveform was divided into three phases. The first phase was used for erasing the original image. The negative voltage duration of 240, 80, 40, and 0 ms were used to drive EPDs continuously when the initial grayscale was “B”, “DG”, “LG”, and “W”, respectively. The grayscale of the original image was driven to white. The second phase was the particle activation phase. A positive voltage of 60 ms was adopted to drive the particle for getting an activation state, and a negative voltage duration of 120 ms was adopted to drive the particle to form a unified reference grayscale according to the driving waveform design of the activation phase. The third phase was used to display a new grayscale. The positive voltage duration of 240, 80, 40, and 0 ms were used to obtain target grayscale of “B”, “DG”, “LG”, and “W”, respectively, and the uniform white reference grayscale was driven to target grayscale, as shown in Figure 7. 

## 4. Experimental Results and Discussion

### 4.1. Testing System

**Figure 8:**
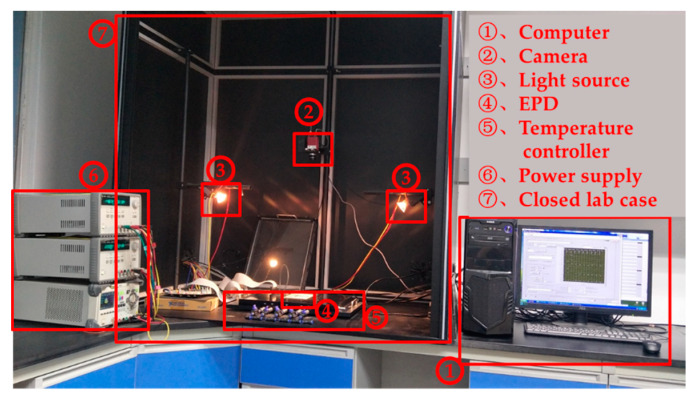
Optical testing device for EPDs. Camera was ADIMEC-1600m. Light sources were Brilliantline 12V 20W 36D Halogen. Temperature controller was Julabo FP25-ME. The power supply was an Agilent 3161A three-way output power supply.

**Figure 9:**
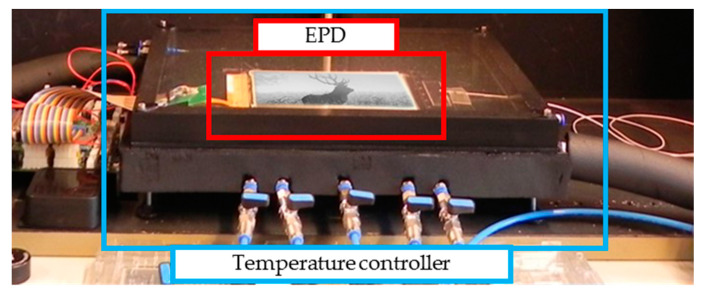
The details of the EPD and the temperature controller. Temperature controller was Julabo FP25-ME.

Microcapsule EPDs (ED060SC7, 6.0 inch, screen resolution is 600 × 800) produced by E-ink (Holding Inc., Hsinchu, Taiwan) were introduced for the experiments in this study. The experimental equipment comprised mainly into two parts: the driving waveform editing system and the brightness data acquisition system. A driving waveform design can be completed by the computer software LabVIEW (LabVIEW10.0.1f3, National Instruments, Austin, TX, USA). The brightness data acquisition system was composed of a driving power supply, a closed lab case, two light sources, a camera, and a temperature controller, as shown in Figure 8. The details of the EPD and the temperature controller are shown in Figure 9. 

A closed lab case was designed to prevent the interference from external light and ensure uniform lighting across the EPD surface. Two light sources were used as the external light source for the data acquisition system. In a confined space, the light environment provided by internal light sources was relatively stable, which provided good comparability for testing the driving effect of driving waveforms. In addition, the electrophoretic motion of particles in an EPD system is easily affected by temperature [31,32], so the experiment temperature was set at 25 °C. 

**Figure 10:**
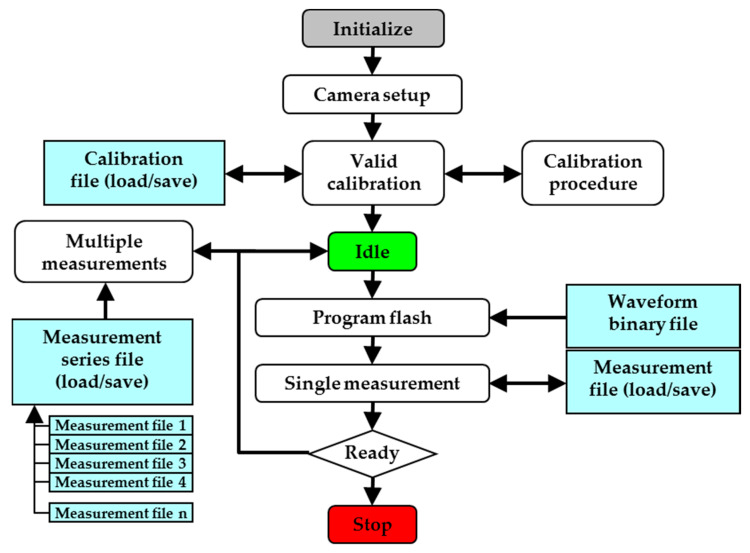
The flow chart of data acquisition. Calibration includes the effective number of measurement area blocks, the international white and black reference grayscale reflectance. The measurement mode includes single measurement and multiple measurement.

The data acquisition system can test the brightness value of EPDs. Firstly, the data acquisition system is initialized, camera parameters are set, and the data acquisition system is calibrated effectively. Then, the system is idled for awaiting calibration results, and the waveform binary file is loaded into the driving system. The system can measure the brightness automatically and export the measurement data. The flow chart of data acquisition is shown in Figure 10. 

**Figure 11:**
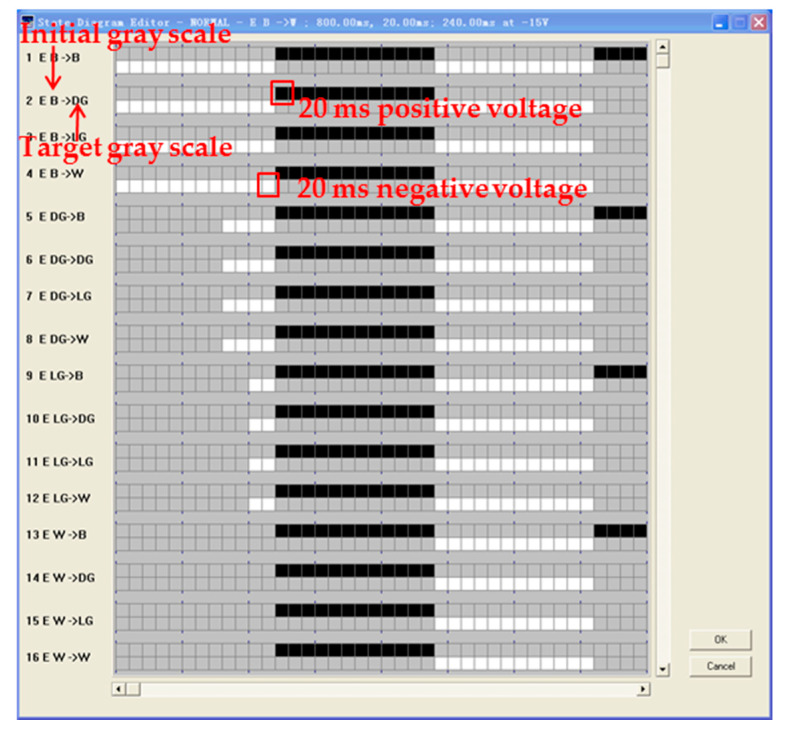
Waveform editing interface. A black grid indicates that a 20 ms positive voltage of +15 V is applied to the pixel electrode, a white grid indicates that a 20 ms negative voltage of −15 V is applied to the pixel electrode, and the rest states are 0 V.

LabVIEW, a software development platform of NI, was applied as a waveform editing system, and the driving waveform can be edited by G language of graphical programming. The waveform editing interface is shown in Figure 11 The designed driving waveform can be converted into a binary file and downloaded directly to the driver circuit. 

### 4.2. Timing Comparison of Driving Waveforms

**Figure 12:**
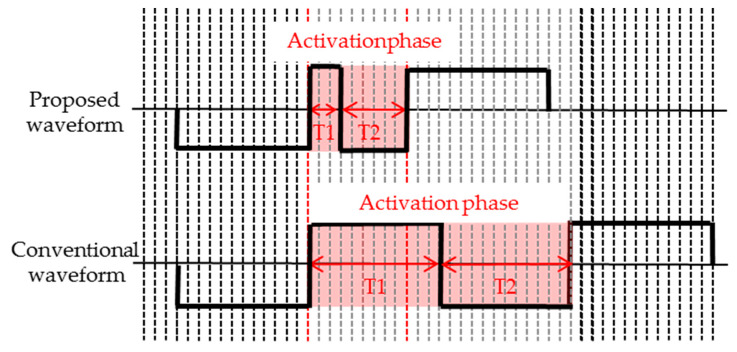
Driving timing comparison of activation phases in driving waveforms, and each vertical line represents 20 ms. In the proposed waveform, T1 is 60 ms and T2 is 120 ms. In the conventional waveform, T1 is 240 ms and T2 is 240 ms.

In different initial grayscale, it only takes 120 ms from the uniform reference grayscale to the white reference grayscale, which is 100 ms less than the conventional driving waveform, whose improving particle activity phase is 60 ms. The activation phase waveform of the proposed driving waveform and the conventional driving waveform is shown in Figure 12.

It can be seen that the activation phase in the proposed driving waveform was shortened by 180 ms, and the uniform reference grayscale phase was shortened by 120 ms. The overall proposed driving waveform was shortened by 300 ms compared to the conventional driving waveform.

### 4.3. Performance Testing

**Figure 13:**
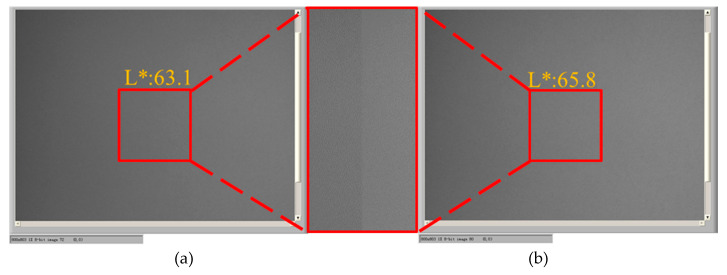
Comparison of the white reference grayscale. (**a**) An EPD was driven to white grayscale by the conventional driving waveform. (**b**) An EPD was driven to white grayscale with the proposed driving waveform.

The conventional driving waveform and the proposed driving waveform were used to drive EPDs to the white state. The results show that the proposed driving waveform can increase the white grayscale by 2.7, as shown in Figure 13. 

**Figure 14:**
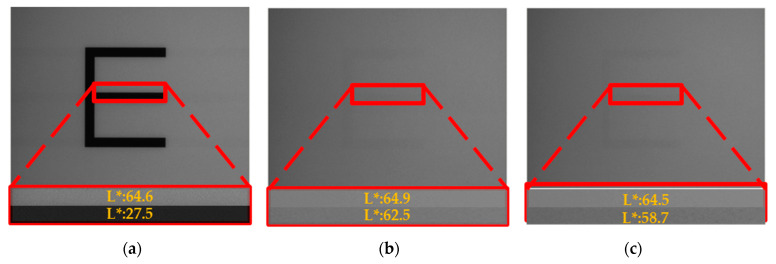
Visibility of the ghost image comparison between the proposed driving waveform and the conventional driving waveform. (**a**) Original image. (**b**) Driving effects of the proposed driving waveform. (**c**) Driving effects of the conventional waveform.

Then, the proposed driving waveform was used to verify the image quality through several experiments. Firstly, a picture “E” was loaded in an EPD, and the proposed driving waveform and the conventional driving waveform were each used to drive it to the white grayscale, so as to explore visibility of the ghost image of each driving waveform, as shown in Figure 14. 

Figure 14a is an original image with a white background and a black font. When it was driven to full white by the proposed driving waveform, the background brightness value of the image was 64.9 nits, and the shadow brightness value was 62.5 nits, so the ghost image brightness was 2.4 nits, as shown in Figure 14b. When it was refreshed to full white by the conventional driving waveform, the background brightness value of the image was 64.5 nits and the shadow brightness value was 58.7 nits, so the ghost image brightness was 5.8 nits, as shown in Figure 14c. Hence, the proposed driving waveform can reduce visibility of the ghost image by 57%. 

The uniformity of the grayscale was analyzed by calculating the change rate of entropy value and the gray value. As shown in Table 2, the proposed driving waveform was used to an EPD, and it can be driven the original image to full white, and the change rates of the entropy value and the gray value were −6.3% and −79.7%, respectively. Then, the conventional waveform was used to test related data, and the change rates of entropy value and gray value were −5.78% and −79.32% respectively. Hence, the proposed driving waveform can reduce the image grayscale texture by 0.52% and improve the image grayscale uniformity by 0.38%. Hence, the proposed driving waveform can optimize the activation phase without affecting the image grayscale quality and reduce the driving duration of 300 ms. 

**Figure 15:**
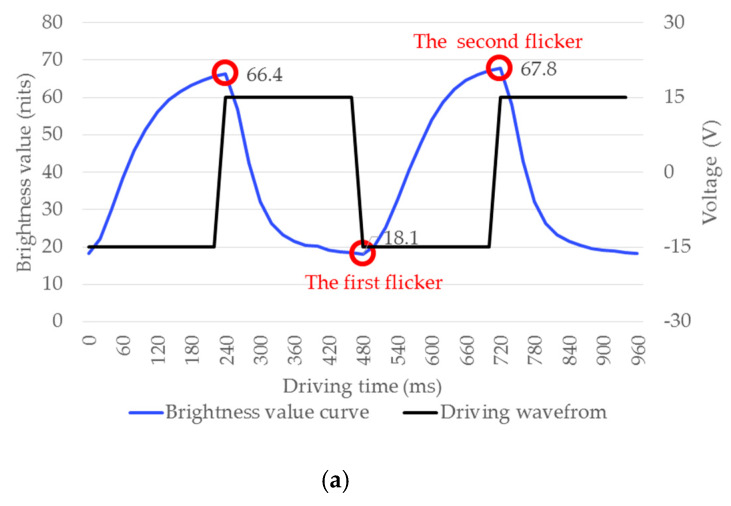

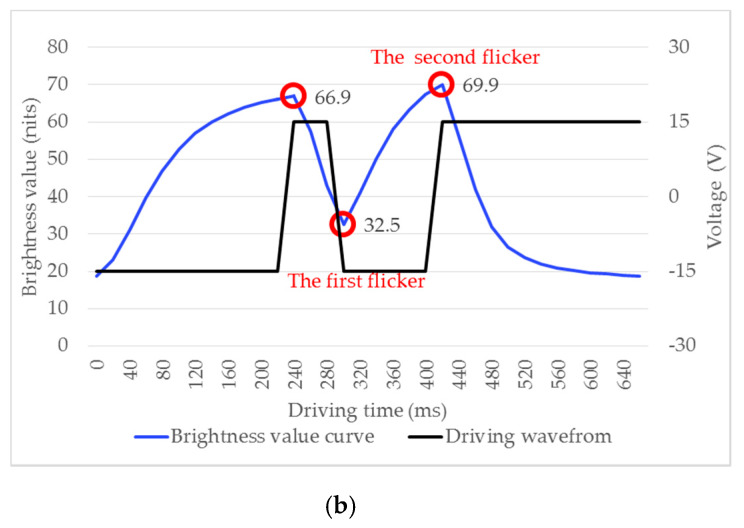
The change curve of EPD brightness. (**a**) When the EPD was driven by the conventional driving waveform. (**b**) When the EPD was driven by the proposed driving waveform.

In addition, the conventional driving waveform has serious flicker when updating an image, especially in the refresh process of B-W. There are three phases in this process: In the erase the image phase, the original image is erased from black to full white. In the particle activation phase, the image needs to be driven to full black and then to full white. In the new image phase, the image is erased from white to full black. The human eye can feel a flicker due to the high contrast switch between black and white, as shown in Figure 15a. However, the proposed driving waveform cannot drive the EPD to full black in the activation phase, so the switching intensity of EPDs can be decreased, resulting in the decrease of the flicker, as shown in Figure 15b.

The extreme values of the brightness curve in the activation phase were 66.4, 18.1, and 67.8 nits, respectively, when the EPD was driven by the conventional waveform, so flickers with the intensity of 48.3 and 49.6 nits were respectively formed. When the EPD was driven by the proposed driving waveform, the extreme values of the brightness curve in the activation phase was 66.9, 32.5, and 69.9 nits, respectively, so flickers with the intensity of 34.4 and 37.4 nits were respectively formed. Therefore, the proposed driving waveform can reduce the flicker intensity by 28.8% and 24.6%, and the overall flicker intensity by 26.7%, as shown in Table 3.

## 5. Conclusions

In this study, a fast response driving waveform for EPDs based on optimized activation phase was proposed by analyzing the characteristics of EPDs. The duration of the driving waveform can be effectively shortened by 300 ms compared to the conventional driving waveform, and the reference white grayscale can be increased by 2.7 at the same time. In addition, it can reduce visibility of the ghost image by 57%, which can improve display quality, and the flicker intensity can be reduced by 26.7% for enhancing the visual experience of human eyes. 

## Figures and Tables

**Table 1:** Change value of EPD brightness in T2.

T1 Durations	40 ms	45 ms	50 ms	55 ms	60 ms	Conventional Waveform
Time for reaching reference grayscale (ms)	120	120	120	120	120	220
Initial brightness values (nits)	33.93	32.81	31.79	30.66	28.13	19.85

**Table 2:** The change of entropy and gray value.

Image Type	Entropy	Change Rate of Entropy (%)	Gray Value	Change Rate of Gray Value (%)
Original image	5.71	–	44.98 × 10^7^	–
Proposed driving waveform	5.35	−6.30	9.13 × 10^7^	−79.70
Conventional driving waveform	5.38	−5.78	9.30 × 10^7^	−79.32

**Table 3:** The change in flicker intensity.

Waveform Type	Intensity of the First Flicker (nits)	Intensity of the Second Flicker (nits)	Total
Conventional driving waveform	48.3	49.6	97.3
Proposed driving waveform	34.4	37.4	41.8
Reduction rate of flicker intensity (%)	28.8	24.6	26.7
